# Evaluation of Antioxidant Activity and Acute Toxicity of *Clausena excavata* Leaves Extract

**DOI:** 10.1155/2014/975450

**Published:** 2014-12-24

**Authors:** Shaymaa Fadhel Abbas Albaayit, Yusuf Abba, Rasedee Abdullah, Noorlidah Abdullah

**Affiliations:** ^1^Institute of Biological Sciences, Faculty of Science, University of Malaya, 50603 Kuala Lumpur, Malaysia; ^2^Department of Biology, College of Science, University of Baghdad, Baghdad, Iraq; ^3^Department of Pathology and Microbiology, Faculty of Veterinary Medicine, Universiti Putra Malaysia, 43400 Serdang, Selangor, Malaysia

## Abstract

*Clausena excavata* (Lour.), locally known as “Kemantu hitam,” is a common plant in Malaysian folklore medicine. This study evaluated the antioxidant properties of the solvent extracts of *C. excavata* leaves and determined the acute toxicity of methanolic extract *C. excavata* (MECE) leaves in Sprague-Dawley rats. Harvested leaves were dried and subjected to solvent extraction using petroleum ether, chloroform, ethyl acetate and methanol in succession. The antioxidant activity of each extract was determined using the ferric-reducing antioxidant power (FRAP) and 2,2-diphenyl-1-picryl dihydrazyl (DPPH) radical scavenging activity. The total phenolic content (TPC) and total flavonoids content (TFC) were estimated by Folin-Ciocalteu and ethanolic aluminium chloride method, respectively. The chloroform extract was found to be highest in flavonoid content, while the methanolic extract showed the highest TPC and antioxidant activity. There was no mortality in rats treated with MECE leaves even at a high dose of 5000 mg/kg body weight. However, the MECE leaves produced mild to moderate pathological changes in the liver and kidneys, shown by mild degenerative changes and leucocyte infiltration. The extract did not affect the haematological parameters or relative weights of the liver or kidneys. Overall, the MECE leaves have potent antioxidant activity and are presumed safe to be used orally as health-promoting product at low to moderate doses.

## 1. Introduction

Free radicals are harmful by-product of cellular oxidative phosphorylation and energy production. These radicals damage various intracellular macromolecules to include DNA, protein, and lipids [1]. Antioxidants have the ability to prevent oxidative damage and inhibit inflammatory conditions by nullifying the activities of free radicals [2]. Currently, the search for plant sources of antioxidants is gaining momentum with* Clausena excavata* (Rutaceae family) among the plants targeted.* C. excavata* is a medicinal plant widely distributed in Southeast Asia and is known by unique local names, such as Chamat in Thailand and Jia huang pi in China. In Malaysia the plant is locally known as “Cherek hitam” and “Kemantu hitam.” The leaves of the plant are used in folklore medicine for the treatment of several illnesses such as malaria, headache, abdominal pain, dysentery, pulmonary tuberculosis, diarrhoea, cold, wound, snake-bite, and poisoning. Recent studies showed that the plant also possessed immune-modulatory [3], analgesic [4], anti-inflammatory, antivirus, anticancer [5], antioxidant [6], antimycobacterial [7], and antifungal [8] activities.* C. excavata* has been reported to exhibit one of the highest beneficial biological activities among* Clausena* genus [9]. These activities are attributed to its high phenolic compounds such as furanocoumarins and flavonoids. The plant also contains other pharmacologically active compounds like coumarin, carbazole alkaloid, and flavonoid glycosides [7, 10–12].

It is imperative that compounds from plants for human use are screened for potential toxicity, particularly for repeated and prolonged applications. Toxicity following overdose of active principles from plant candidates poses limitations to its use, since it may result in deaths in some cases [13]. Although a recent study reported that repeated oral doses of the methanol extract of* C. excavata *stem [14] did not cause significant pathological changes in rats, there is currently a paucity of information on the safe use of the plant leaves. This present study aims to determine the antioxidant properties and the safety of oral application of methanol extract of* C. excavata *leaves in rats.

## 2. Materials and Methods

### 2.1. Collection, Identification, and Processing of Plant Material

*C. excavata* leaves were collected from Pendang, Kedah, Malaysia. The plant was identified and authenticated by Dr. Shamsul Khamis of the Biodiversity Unit, Institute of Bioscience, Universiti Putra Malaysia. The leaves were sorted, washed, and dried at room temperature for two weeks before being grinded into powder and stored in air tight plastic bags.

### 2.2. Extraction of Plant Material

Extraction was done at room temperature using petroleum ether, chloroform, ethyl acetate, and methanol in succession. The extraction at a 1 : 5 dried plant weight to volume ratio began with petroleum ether for 3 days. The filtrate was collected and the residues were subject to further extraction with chloroform, ethyl acetate, and methanol, and the filtrate was collected after each solvent extraction. All filtrates were evaporated to dry under reduced pressure using rotary evaporator at 45 to 50°C to obtain crude extracts.

### 2.3. *In Vitro* Assessment of Antioxidant Activity

#### 2.3.1. 2,2-Diphenyl-1-picryl Dyhydrazyl Free Radical Scavenging Method

DPPH scavenging activity of* C. excavata* petroleum ether, chloroform, ethyl acetate, and methanol extracts were estimated using the method of Lim and Murtijaya [15] and Alnajar et al. [16]. Briefly, 195 *μ*L DPPH reagent was added to 5 *μ*L of samples or quercetin standard. The mixtures were vigorously shaken and incubated in the dark for 2 hours at room temperature. Absorbance reading was done using a spectrophotometer at 515 nm against a blank.

#### 2.3.2. Ferric Reducing Antioxidant Power Assay Method

Ferric-reducing antioxidant power (FRAP) of each fraction was estimated according to the method of Alnajar et al. [16] and Mayakrishnan et al. [17]. Briefly, the freshly prepared reaction mixture containing 25 mL of acetate buffer, 2.5 mL of 2,4,6-Tripyridyl-s-Triazine (TPTZ) solution and 2.5 mL of FeCl_3_ *·*6H_2_O was incubated for 5 minutes at 37°C water-bath and absorbance was measured using a spectrophotometer at 593 nm. Thirty microliter of extract and ninety microliter of distilled water were added to nine hundred microliters of FRAP working reagent and mixed before absorbance was measured at 593 nm.

#### 2.3.3. Total Phenol Content

The total phenol content (TPC) of each fraction was determined as described earlier using Folin-Ciocalteu reagent and Gallic acid as a standard [16, 18]. Absorbance was measured spectrophotometrically at 750 nm and results were expressed as mg Gallic acid equivalent (GAE) per g of extract (mg GAE/g).

#### 2.3.4. Total Flavonoid Content

Total flavonoid was estimated using the method developed by Adedapo et al. [19]. Briefly, 0.5 mL of the sample was added to 0.5 mL of 2% AlCl_3_ dissolved in ethanol. After incubation for one hour at room temperature, the absorbance was measured at 420 nm. A calibration curve was prepared using (+) quercetin equivalent per gram (QE/g) extract.

#### 2.3.5. Phytochemical Characterization of the Methanolic Extract of* C. excavata*

The LC-MS/MS analysis was carried out as described earlier by Mayakrishnan et al. [18], using an AB Sciex 3200 Q trap with Perkin Elmer UHPLC FX15.

### 2.4. Experimental Animals

Thirty-six adult male and female Sprague-Dawley rats, 8 to 10 weeks old, weighing between 165 and 200 g, were procured from the Animal House of the Faculty of Medicine, University of Malaya, Malaysia. The rats were maintained in clean stainless steel cages at an ambient temperature of 25 ± 2°C and fed standard rat/mouse pellet (Specialty Feeds, Glen Forrest, Western Australia) and water* ad libitum *for one week before experimentation. All studies performed were approved by the Institutional Animal Care and Use Committee, University of Malaya (ISB/22/007/2013/1111/SFA).

#### 2.4.1. Animal Grouping and Acute Oral Toxicity Study

The Animals were subdivided into 2 groups, males (*n* = 16) and females (*n* = 16). The groups were designated as control male (CM), control female (CF), given 10% Tween-20 orally, and treatment groups, administered extract orally as follows: male 1 (M1, 2000 mg/kg body weight extract), male 2 (M2, 5000 mg/kg body weight extract), female 1 (F1, 2000 mg/kg body weight extract), and female 2 (F2, 5000 mg/kg body weight extract). The acute oral toxicity of the methanolic extract* C. excavata* (MECE) leaves was evaluated following Organization for Economic Cooperation and Development (OECD-423) guidelines [20] with slight modification. The M1 and F1 rats were each administered one dose of 2000 mg/kg orally and M2 and F2 rats were administered 5000 mg/kg body weight of the extract suspended in 10% Tween-20 (5 mL/kg). The rats were monitored for mortality for 48 hours and humanely euthanized after 14 days.

#### 2.4.2. Blood Parameters

The rats were fasted overnight with free access to water. Blood samples were collected in plain and EDTA coated tubes. Serum was separated by centrifugation of whole clotted blood at 3000 r/min (Hettich, EBA 20) for 10 minutes and used to determine concentrations of alkaline phosphatase (ALP), aspartate aminotransferase (AST), alanine aminotransferase (ALT), albumin, bilirubin, creatinine (Cr), urea (BUN), sodium (Na^+^), potassium (K^+^), and chloride (Cl^−^). Non-coagulated blood samples collected in EDTA tubes were used for hematological evaluation; red blood cell count (RBC), hemoglobin (Hb) concentration, and white blood cell count (WBC).

### 2.5. Histopathology

All the rats were humanely sacrificed and the gross pathology of internal organs was evaluated. Tissue samples of the liver and kidney were collected from all the experimental groups, weighed, and then fixed in 10% buffered formalin. The fixed tissues were embedded, sectioned, and stained with hematoxylin and eosin stain and observed under light microscopy.

### 2.6. Statistical Analyses

All data obtained from this study were analysed using SPSS computer statistical software package, version 19, and expressed as mean ± standard deviation (SD). Significance within groups was determined using one-way ANOVA and Tukey's posttest analysis at *P* < 0.05 for significance.

## 3. Results

Extraction of air-dried ground* C. excavata* leaves with petroleum ether, chloroform, ethyl acetate, and methanol produces extract yield (wt/wt) of 1.56, 2.57, 0.38, and 0.94%, respectively.

### 3.1. Ferric Reducing Antioxidant Power

**Figure 1:**
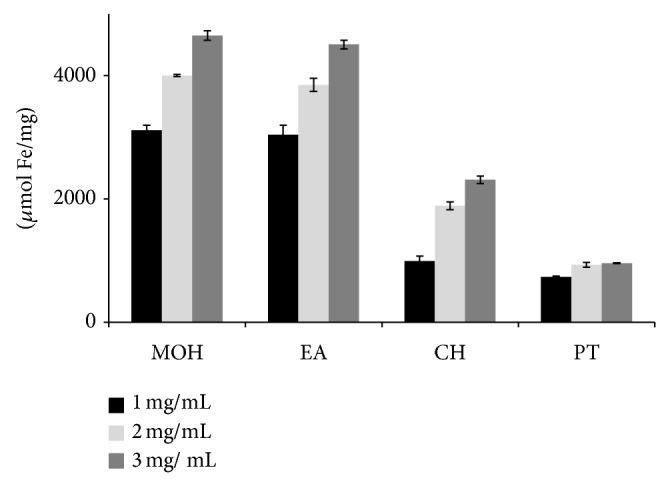
Ferric-reducing antioxidant power of* Clausena excavata *extracts in solvents MOH = methanol; EA = ethyl acetate; CH = chloroform; PT = petroleum ether.

The increase in FRAP absorption suggested that the extract has free radical scavenging properties. The study showed that methanolic and ethyl acetate extracts at 3 mg/mL possessed the highest antioxidant activity (Figure 1).

### 3.2. DPPH Free Radical Scavenging of* Clausena excavata *Extracts

**Figure 2:**
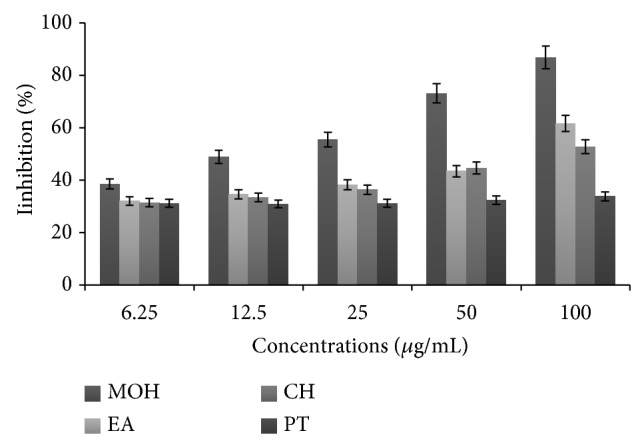
Scavenging activity of* Clausena excavata *extracts on DPPH radical. MOH = methanol; EA = ethyl acetate; CH = chloroform; PT = petroleum ether.

The DPPH radical scavenging activities of different extracts of* C. excavata* leaves are presented in Figure 2. The study showed that the methanolic extract possessed the highest radical scavenging activity.

### 3.3. Total Phenolic and Flavonoid Contents of* Clausena excavata* Extracts

Table 1 shows the phenolic and flavonoid content of the different crude leave extracts. The MECE leaves had the highest amount of phenolic compounds, while the chloroform extract had the highest total flavonoid content.

### 3.4. Phytochemical Characterization of the Methanolic Extract of* C. excavata*

Based on LCMS/MS analysis, the MECE contains pipecolic acid, myricetin glucoside conjugate, quercetin-rhamnose-hexose-rhamnose, kaempferol conjugate, phenolic acid, flavonoids, furocoumarin, and 8-geranyloxy psoralen (Table 2).

### 3.5. Acute Oral Toxicity Study

There were no mortality or behavioural abnormality in rats treated orally with 2000 and 5000 mg/kg body weight of the methanolic fraction of the plant extract showing that the oral LD50 of MECE leaves was greater than 5000 mg/kg body weight. Metabolic studies showed that body weight was lower in the group treated with high doses of extract. The kidneys and liver of rats treated with the extract did not show gain in relative organ weights (Table 3).

### 3.6. Serum Biochemistry and Hematology

#### 3.6.1. Liver Function Test

The liver function tests showed slightly increased serum enzyme concentrations in both male and female rats fed MECE leaves. M2 had elevated serum ALP, AST, and ALT concentrations, while in F2, only the ALT and ALP concentration increased slightly (*P* < 0.05). Total serum bilirubin concentration increased in both F1 and F2 rats (Table 4).

#### 3.6.2. Renal Function Test

Serum creatinine concentrations of M1 and M2 rats were significantly (*P* < 0.05) lower than that of NC male rats. However, the BUN concentration of the M2 rats were not significantly (*P* > 0.05) different from that of the control. The mean BUN concentration of F2 rats was significantly (*P* < 0.05) higher than that of control by 24%. Similarly, serum Na^+^, K^+^, and Cl^−^ concentrations were variably elevated among the male and female treatment groups (Table 5).

#### 3.6.3. Hematological Parameters

The erythrocyte and leukocyte counts and Hb concentration did not significantly (*P* > 0.05) vary between the control and experimental rats following administration of* C. excavata *extract (Table 6).

### 3.7. Histopathology

**Figure 3:**
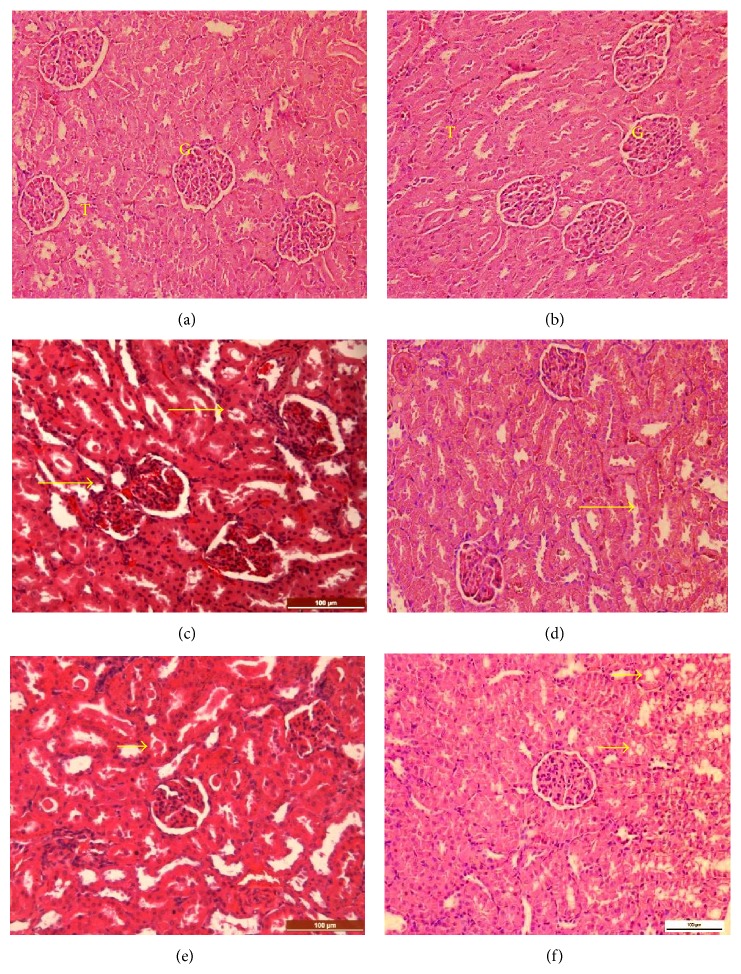
Kidney after treatment with methanolic extract of* Clausena excavata*. (a) and (b) Kidney (control; male and female) showing normal glomeruli (G) and tubules (T) in the cortex region. (c) and (d) Kidney (low dose; 2000 mg/kg, male and female rats) showing tubular degeneration and mild mononuclear cell infiltration (arrows). (e) and (f) Kidney (high dose; 5000 mg/kg, male amd female rats) showing presence of cast in the tubular lumen and mild tubular degeneration (H&E, ×200).

Microscopic evaluation of tissue sections of M1 and M2 rats did not show abnormal histopathological changes. However, kidney sections of F1 and F2 rats showed histopathological changes that varied according to dose level. These changes include mild tubular epithelial degeneration, increased bowman's capsular space as a result of constriction of the glomerulus, mild congestion, cast-like materials within the renal tubular spaces, and mild leukocytic infiltration typified by lymphocyte, and neutrophil infiltration in the renal interstitium (Figure 3).

**Figure 4:**
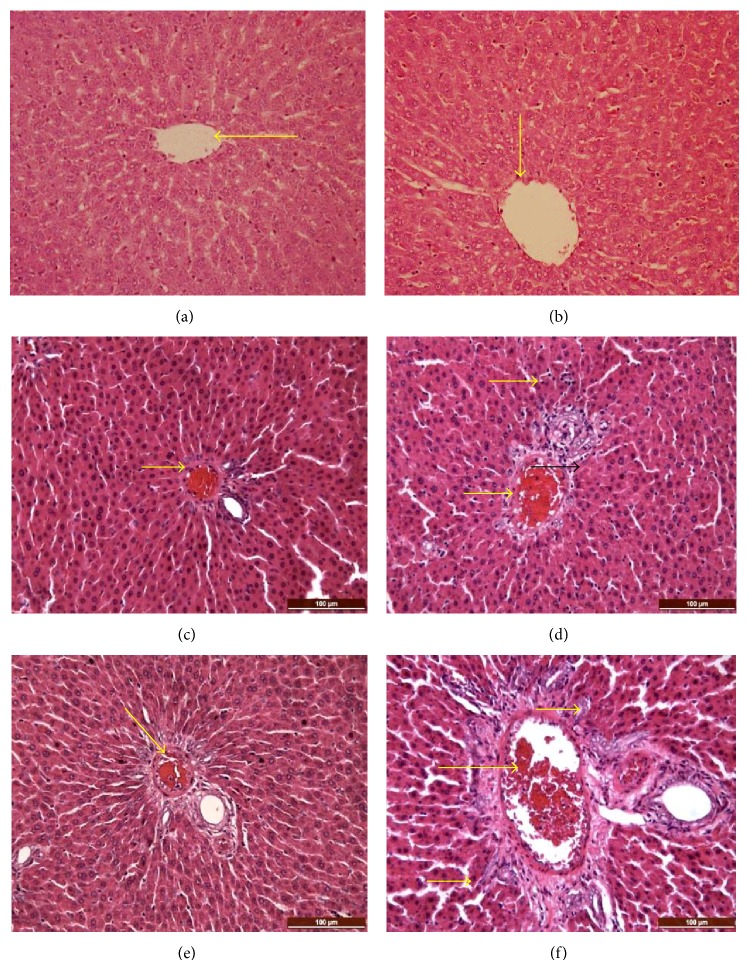
Liver treated with methanolic extract of* Clausena excavata*. (a) and (b) Normal hepatocytes, parenchyma, and central vein, respectively. (c) and (d) Liver (low dose; 2000 mg/kg, male and female rats) showing mononuclear cell infiltrations and congestion of the central vein (arrows). (e) and (f) Liver (high dose; 5000 mg/kg, male and female rats) showing infiltration of kupffer cells, vascular congestion, and mild periportal necrosis. (H&E, ×200).

Microscopic examination of the liver sections of M1, F1 and M2, F2 rats showed central vein congestion, cloudy hepatocytes swelling, and diffuse infiltration by Kupffer cells. Multifocal areas of mild perilobular necrosis coupled with neutrophil infiltration were observed in some sections of the liver tissue of the male rats given a high dose of extract, indicating that the effect of the high dose MECE leaves was more severe in the male than female rats (Figure 4).

## 4. Discussion

Over the past years, a handful of pharmacologically active constituents of plants belonging to the family Rutaceae have been identified, these include methoxyflavones, furoquinoline alkaloids, furanone-coumarins, and flavonoids. These constituents were shown to have vital effects on inflammation, viral infections, and digestive system disorders such as gastritis and enteritis [25–27]. Phytochemical screening showed that these plants contain furanone-coumarins, flavonoids, and glycosides [12, 28].* C. excavata*, in particular, possesses a wide range of pharmacological properties which range from antioxidant properties [6], hyperglycemic, anti-rhinitis, anti-inflammatory [29], anti-nociceptive [4], anti-viral, anti-cancer and anti-fungal activities [8, 30]. To determine the antioxidant activities of* C. excavata*, most of these studies used the bark and stem of the plant, not the leaves. In the present study, the MECE leaves were observed to strongly reduce FeCl_3_ and showed the scavenging activity for DPPH, at potency close to that of quercetin. This observation suggests that the* C. excavata* extract, by its phenolic and flavonoid contents, may be able to donate electrons to the free radicals [31, 32]. It is well-documented that phenolic compounds are among principal and effective antioxidant constituents in plant foods [33]. Thus quantification of phenolic contents of an extract would be essential to determine its antioxidant activity. Previous studies have reported that MECE leaves with high phenolic content inhibit lipid peroxidation [6, 29]. Flavonoids of the MECE leaves with their wide spectrum of medicinal actions are the major polyphenols in the scavenging of oxidizing species like hydroxyl radical, superoxide anion, or peroxyl radicals [34] (Alnajar et al., 2012). The chloroform extract of* C. excavata *was shown in the current study to have the highest concentration of flavonoids, which is comparable with those reported by others [35, 36]. Thus it is not surprising that* C. excavata*, with its potent phenolic antioxidant, is effective in the treatment of various oxidative stress-related chronic diseases.

The MECE leaves showed good antioxidant activity and were subsequently selected for phytochemical screening and animal studies. Initial investigation using LCMS/MS confirmed that the MECE leaves are rich in phenolic/flavonoids-based compounds [11]. Notably, these compounds in the MECE leaves possess several biological activities; for example, coumarin has been used as an anti-inflammatory, anticholinesterase, antioxidant, and anticancer compound [25, 27, 37]; quercetin glycoside has been used as a hepatoprotective compound in CCl_4_-induced hepatic damage, while kaempferol and myricetin glucosides have been shown to have high anti-inflammatory and antioxidant activities [38, 39].

The toxicity of MECE leaves in rats was evaluated by an acute 14-day study. No mortality or symptom of toxicity was observed in the rats in the study. The LD50 of MECE leaves in both sexes of rats were greater than 5000 mg/kg body weight. This is in accordance with an earlier study [14], which reported that oral LD50 of the methanolic extract from the stem of* C. excavata* is more than 5000 mg/kg body weight in both sexes in rats. Therefore, the high LD50 of MECE leaves indicates that the extract is relatively safe, especially for oral administration. According to Ecobichon [40] and OECD, any test compound that does not produce adverse effects at a dose exceeding 5 g/kg body weight is considered nontoxic.

Change in body weight and relative organ weight of animals is an indicator of adverse effect of administered drugs or chemicals [41, 42]. The organs of these animals tend to swell or become damaged, which will subsequently alter their organ-to-body weight ratios upon exposure to toxic substances. In this study, the body weights of rats (male and female) treated with MECE leaves increased gradually but insignificantly (*P* > 0.05) compared with the control rats. Similarly, the relative organ weights showed no significant (*P* > 0.05) difference between test and control groups.

Increased serum creatinine is a good indicator of abnormal kidney function while simultaneous increases in serum AST and ALT concentrations are associated with hepatopathy [43]. Our study showed significant (*P* < 0.05) but variable increases in serum enzymes in male and female rats treated with MECE leaves. Serum bilirubin concentration was significantly (*P* < 0.05) elevated in MECE leaves-treated female rats only. In this case, increase in ALP level is associated with increased hepatic synthesis, in the presence of increasing biliary pressure and obstruction in the normal bile flow as a result of damage or inflammation [36]. In male rats treated with low dose MECE leaves, we observed that the serum bilirubin and ALP concentrations were not significantly (*P* > 0.05) different from that of the controls, an indication that the liver has not been compromised by the treatment.

Significant increase in BUN without abnormality in serum creatinine concentration in female rats treated with a high dose MECE leaves is indicative of hemoconcentration and/or high dietary protein rather than kidney impairment [44, 45]. This assertion is based on the fact that serum creatinine is a more accurate marker of glomerular filtration and kidney function than urea. Serum Na^+^ was slightly elevated in both treated male and female rats, while K^+^ was elevated in the male rats only and Cl^−^ in female rats treated with high dose MECE leaves only. Since the concentrations of these electrolytes in blood are generally governed by diet and water intake, these changes do not reflect adverse effect of MECE leaves.

Although MECE leaves-treatment of rats produced mild to moderate pathological changes in the kidneys and liver, neither mortality nor symptoms of toxicity at the doses used in this study were observed; thus the extract is presumed safe for oral application.

## 5. Conclusion

The MECE leaves contain pharmacologically active compounds including flavonoids and phenolics, which are good antioxidants. The MECE leaves did not exhibit obvious toxicity at concentrations even at a high dose of 5000 mg/kg body weight. Thus the MECE leaves have potent antioxidant activity and are presumed safe to be used orally as health-promoting product at low to moderate doses.

## Figures and Tables

**Table 1:** Total phenolic and flavonoid contents of *Clausena excavata* extracts.

Extract content	Solvent			
	Methanol	Ethyl acetate	Chloroform	Petroleum ether
TPC (mg GAE/g extract)	522.0^a^ ± 11.6	497.0^b^ ± 7.1	373.0^c^ ± 6.9	157.0^d^ ± 7.5
TFC (mg QE/g extract)	96.7^c^ ± 2.0	142.5^b^ ± 1.0	188.6^a^ ± 3.0	25.8^d^ ± 0.8

Values are mean ± SD.

^a,b,c,d^Mean ± SD with different superscripts are significantly different at *P* < 0.05.

TPC = total phenolic content; TFC = total flavonoid content; GAE = Gallic acid equivalent; QE = quercetin equivalent.

**Table 2:** Compound identified from MECE via LCMS/MS analysis.

Retention time	Molecular weight	MS/MS fragments ions [M − H]^−^, [M + H]^+^	Tentative Identification	References
3.69	756.2	(−) 755, 300, 271.0, 255.1, 179.0, 151.0	Quercetin-rhamnose-hexose-rhamnose	Chua et al., 2011 [21] Kachlicki et al., 2008 [22] Del Rio et al., 2004 [23]

3.9	772.2	(−) 771, 316, 271.1, 179.0, 151.0	Myricetin glucoside Conjugate Myricetin 3-O-rhamnosyl-glucoside 7-O-rhamnoside	Chua et al., 2011 [21] Kachlicki et al., 2008 [22] Del Rio et al., 2004 [23]

4.3	740.2	(−) 739.2, 284.1, 255.0, 179, 151.0, 179.0,	Kaempferol conjugate	Chua et al., 2011 [21] Zhao et al., 2011 [24]

7	179.3	(+) 161.2, 135.1, 133.1	Caffeic acid	Chua et al., 2011 [21]

6.72		(+) 77.0, 91.0, 95.0, 105.0, 119.1, 134.1, 148.3, 149.2, 156.3, 161.3, 174.4, 177.3, 179.3, 190.4, 202.4, 204.4, 206.4, 218.4, 221.4, 230.4, 232.4, 243.4, 245.4, 246.4, 256.4, 270.4, 284.4, 288.4, 298.4, 316.4, 326.4, 344.4, 362	8-Geranyloxy psoralen	

5.95		(+) 77.91, 91.0, 95.0, 111.0, 119.0, 133.1, 147.2, 149.2, 161.2, 177.2, 179.3, 191.3, 203.3, 243.3, 245.4, 247.3, 255.3, 271.4, 273.4, 289.4, 291.4, 313.4, 323.4, 331.4, 341.4, 359.4, 377.4	Unidentified furocoumarin	

5.27		(+) 117.1, 134.2, 146.2, 147.2, 150.2, 160.3, 163.3, 180.3, 202.3, 246.4, 473.4, 491.5, 509.5, 527.6	Unidentified flavonoids	

5.5		(+) 119.0, 134.1, 146.2, 147.2, 162.3, 164.3, 180.3, 201.3, 229.3, 245.3, 240.4, 246.4, 294.4, 341.3, 404.4, 491.4, 523.3, 531.5, 541.6, 559.6	Unidentified flavonoids	

6.2		(+) 146.2, 179.3, 194.4, 203.3, 247.3, 292.4, 324.4, 324.4, 370.4, 388.4, 470.4, 557.6, 735.7	Unidentified flavonoids	

4.72		(−) 107.0, 123.0, 137.1, 139.1, 163.1, 179.1, 195.2, 197.2, 241.1	Unidentified phenolic acid	

**Table 3:** Relative weights of selected organs after administration of a single dose methanolic extract of *Clausena excavata*.

Organ	Sex	MECE treatment (mg/kg body weight)		
		Untreated	2000	5000
		Relative organ weight (g)		
Liver	Male	2.33 ± 0.10	2.55 ± 0.19	2.58 ± 0.20
	Female	2.60 ± 0.07	2.78 ± 0.14	2.82 ± 0.16

Kidney	Male	0.68 ± 0.01	0.74 ± 0.02	0.70 ± 0.01
	Female	0.55 ± 0.01	0.61 ± 0.02	0.63 ± 0.03

Values are mean ± SD.

MECE = methanolic extract *Clausena excavata*; untreated = rats given normal diet.

**Table 4:** Effect of methanolic extract of *Clausena excavata *extract on liver biochemical parameters.

Treatment group (MECE leaves)	ALT (U/I)	AST (U/I)	ALP (U/I)	Albumin (g/dL)	Bilirubin (*µ*mol/L)
Male					
CM	57.0^a^ ± 2.2	196.0^a^ ± 3.6	177.7^a^ ± 7.5	12.2^a^ ± 0.3	2.19^a^ ± 0.01
M1 (2000 mg/kg b.wt)	59.1^a^ ± 2.3	183.4^a^ ± 11.7	228.2^b^ ± 27.0	13.0^b^ ± 0.3	2.73^b^ ± 0.17
M2 (5000 mg/kg b.wt)	64.7^b^ ± 4.3	256.6^b^ ± 20.4	248.6^b^ ± 23.4	13.8^b^ ± 0.3	2.42^a^ ± 0.22
Female					
CF	46.8^a^ ± 2.5	165.1^a^ ± 6.2	143.3^a^ ± 10.7	14.7^a^ ± 0.7	1.92^a^ ± 0.03
F1 (2000 mg/kg b.wt)	47.8^a^ ± 2.1	181.6^a^ ± 15.9	109.0^b^ ± 5.2	15.2^a^ ± 0.2	2.76^b^ ± 0.19
F2 (5000 mg/kg b.wt)	53.7^b^ ± 5.2	195.1^a^ ± 26.4	193.2^c^ ± 19.3	14.8^a^ ± 0.3	2.83^b^ ± 0.16

Values are mean ± SD.

^a,b,c^For each sex, means within column with different superscript are significant at *P* < 0.05.

ALT = alanine transaminase; AST = aspartate transaminase; ALP = alkaline phosphatase.

MECE = methanolic extract *Clausena excavata*; CM = untreated control male; CF = untreated control female; M1, M2, F1, and F2 are treatment groups; b.wt = body weight.

**Table 5:** Effects of methanolic extract of *Clausena excavata *on kidney biochemical parameters.

Treatment Group (MECE leaves)	Sodium (mmol/L)	Potassium (mmol/L)	Chloride (mmol/L)	Urea (mmol/L)	Creatinine (umol/L)
Male					
CM	144.31^a^ ± 0.7	4.90^a^ ± 0.10	104.8^a^ ± 0.1	6.42^a^ ± 0.34	635.6^a^ ± 21.4
M1 (2000 mg/kg b.wt)	145.8^b^ ± 0.2	4.66^b^ ± 0.06	103.6^b^ ± 1.0	4.61^b^ ± 0.21	379.6^b^ ± 48.7
M2 (5000 mg/kg b.wt)	146.2^b^ ± 0.4	5.00^b^ ± 0.25	105.0^a^ ± 0.4	5.90^c^ ± 0.21	415.5^b^ ± 25.7
Female					
CF	144.4^a^ ± 0.7	4.70^a^ ± 0.06	104.3^a^ ± 1.1	5.79^a^ ± 0.17	576.8^a^ ± 31.1
F1 (2000 mg/kg b.wt)	144.2^b^ ± 0.7	4.64^a^ ± 0.29	104.4^a^ ± 0.4	5.81^a^ ± 0.24	424.6^b^ ± 19.3
F2 (5000 mg/kg b.wt)	146.1^b^ ± 0.9	4.90^a^ ± 0.15	106.8^b^ ± 0.6	7.70^b^ ± 0.29	561.6^a^ ± 29.8

Values are mean ± SD.

^a,b,c^For each sex, means within column with different superscript are significant at *P* < 0.05.

MECE = methanolic extract *Clausena excavata*; CM = untreated control male; CF = untreated control female; M1, M2, F1, and F2 are treatment groups; b.wt = body weight.

**Table 6:** Effects of methanolic extract of *Clausena excavata *extract on hematological parameters in rats.

Parameter	Sex	MECE leaves treatment (mg/kg body weight)		
		Untreated	2000	5000
		Relative organ weight (g)		
Erythrocyte (10^12^/L)	Male	8.44 ± 0.11	8.04 ± 29.00	8.32 ± 0.20
	Female	7.80 ± 0.03	7.79 ± 0.03	7.77 ± 0.02

Leukocyte (10^9^/L)	Male	8.46 ± 0.21	9.94 ± 0.37	9.62 ± 0.10
	Female	6.55 ± 0.96	6.20 ± 0.71	6.13 ± 0.34

Hemoglobin (g/L)	Male	13.80 ± 0.20	14.02 ± 0.31	13.40 ± 0.07
	Female	13.10 ± 0.12	12.63 ± 0.48	12.50 ± 0.27

Values are mean ± SD.

MECE = methanolic extract *Clausena excavata*; untreated = rats given normal diet without MECE leaves treatment.

## Abbreviations

MECE: Methanolic extraction* Clausena excavata*
ALP: Alkaline phosphatase
ALT: Alanine aminotransferase
AST: Aspartate aminotransferase
Cr: Creatinine
BUN: Blood urea nitrogen
OECD: Organization for Economic Cooperation and Development
CDL: Clinical Diagnostic Laboratory
UMMC: University of Malaya Medical Centre
H&E: Haematoxylin and eosin
ANOVA: Analysis of variance
SD: Standard deviation.

## References

[1] Harman D. (1956). Aging: a theory based on free radical and radiation chemistry. *The Journal of Gerontology*.

[2] Khanna D., Sethi G., Ahn K. S., Pandey M. K., Kunnumakkara A. B., Sung B., Aggarwal A., Aggarwal B. B. (2007). Natural products as a gold mine for arthritis treatment. *Current Opinion in Pharmacology*.

[3] Manosroi A., Saraphanchotiwitthaya A., Manosroi J. (2004). Immunomodulatory activities of fractions from hot aqueous extract of wood from *Clausena excavata*. *Fitoterapia*.

[4] Rahman M. T., Alimuzzaman M., Shilpi J. A., Hossain M. F. (2002). Antinociceptive activity of *Clausena excavata* leaves. *Fitoterapia*.

[5] Sharif N. W. M., Mustahil N. A., Noor H. S. M. (2011). Cytotoxic constituents of *Clausena excavata*. *African Journal of Biotechnology*.

[6] Guntupalli C., Kumar G. S., Kumar A. S., Tubati T. (2012). Evaluation of antioxidant activity of the methanolic leaf extract of *Clausena excavata* Burm. f.(Rutaceae) using the lipid peroxidation model. *Pharmacognosy Journal*.

[7] Sunthitikawinsakul A., Kongkathip N., Kongkathip B. (2003). Coumarins and carbazoles from *Clausena excavata* exhibited antimycobacterial and antifungal activities. *Planta Medica*.

[8] Kumar R., Saha A., Saha D. (2012). A new antifungal coumarin from *Clausena excavata*. *Fitoterapia*.

[9] Arbab I. A., Abdul A. B., Aspollah M., Abdullah R., Abdelwahab S. I., Ibrahim M. Y., Ali L. Z. (2012). A review of traditional uses, phytochemical and pharmacological aspects of selected members of *Clausena genus* (Rutaceae). *Journal of Medicinal Plants Research*.

[10] Shang L.-J., Wen G.-Y., Zhou J., Hao X.-J. (1993). Clausenlactam: a new macrocyclic lactam from *Clausena excavata*. *Acta Metallurgica Sinica*.

[11] Ito C., Itoigawa M., Katsuno S., Omura M., Tokuda H., Nishino H., Furukawa H. (2000). Chemical constituents of *Clausena excavata*: isolation and structure elucidation of novel furanone-coumarins with inhibitory effects for tumor-promotion. *Journal of Natural Products*.

[12] Xin Z.-Q., Lu J.-J., Ke C.-Q., Hu C.-X., Lin L.-P., Ye Y. (2008). Constituents from *Clausena excavata*. *Chemical and Pharmaceutical Bulletin*.

[13] Sani D., Sanni S., Sandabe U. K., Ngulde S. I. (2009). Effect of intake of aqueous stem extract of *Anisopus mannii* on haematological parameters in rats. *International Journal of Applied Research in Natural Products*.

[14] Puongtip K., Ampai P., Narong N., Prapadsorn P., Vichai R. (2011). Acute and repeated dose 90-day oral toxicity studies of *Clausena excavata* extract in rats. *Journal of Pharmaceutical and Biomedical Research*.

[15] Lim Y. Y., Murtijaya J. (2007). Antioxidant properties of *Phyllanthus amarus* extracts as affected by different drying methods. *LWT—Food Science and Technology*.

[16] Alnajar Z. A. A., Abdulla M. A., Ali H. M., Alshawsh M. A., Hadi A. H. A. (2012). Acute toxicity evaluation, antibacterial, antioxidant and immunomodulatory effects of Melastoma malabathricum. *Molecules*.

[17] Mayakrishnan V., Veluswamy S., Sundaram K. S., Kannappan P., Abdullah N. (2013). Free radical scavenging potential of *Lagenaria siceraria* (Molina) Standl fruits extract. *Asian Pacific Journal of Tropical Medicine*.

[18] Mayakrishnan V., Abdullah N., ZainalAbidin M. H. (2013). Investigation of the antioxidative potential of various solvent fractions from fruiting bodies of Schizophyllum commune (Fr.) mushrooms and characterization of phytoconstituents. *Journal of Agricultural Science*.

[19] Adedapo A. A., Jimoh F. O., Koduru S., Afolayan A. J., Masika P. J. (2008). Antibacterial and antioxidant properties of the methanol extracts of the leaves and stems of *Calpurnia aurea*. *BMC Complementary and Alternative Medicine*.

[20] OECD (1998). *OECD Guidelines for Testing of Chemicals*.

[21] Chua L. S., Latiff N. A., Lee S. Y., Lee C. T., Sarmidi M. R., Aziz R. A. (2011). Flavonoids and phenolic acids from *Labisia pumila* (Kacip Fatimah). *Food Chemistry*.

[22] Kachlicki P., Einhorn J., Muth D., Kerhoas L., Stobiecki M. (2008). Evaluation of glycosylation and malonylation patterns in flavonoid glycosides during LC/MS/MS metabolite profiling. *Journal of Mass Spectrometry*.

[23] Del Rio D., Stewart A. J., Mullen W., Burns J., Lean M. E. J., Brighenti F., Crozier A. (2004). HPLC-MSn analysis of phenolic compounds and purine alkaloids in green and black tea. *Journal of Agricultural and Food Chemistry*.

[24] Zhao Y., Chen P., Lin L., Harnly J. M., Yu L., Li Z. (2011). Tentative identification, quantitation, and principal component analysis of green pu-erh, green, and white teas using UPLC/DAD/MS. *Food Chemistry*.

[25] Nakamura T., Kodama N., Arai Y., Kumamoto T., Higuchi Y., Chaichantipyuth C., Ishikawa T., Ueno K., Yano S. (2009). Inhibitory effect of oxycoumarins isolated from the Thai medicinal plant *Clausena guillauminii* on the inflammation mediators, iNOS, TNF-*α*, and COX-2 expression in mouse macrophage RAW 264.7. *Journal of Natural Medicines*.

[26] Guntupalli C., Ramaiah M., Kumar G. S. (2013). RP-HPLC analysis and antimicrobial screening of *Clausena excavata* burm.F . (rutaceae). *International Journal of Phytotherapy*.

[27] Venugopala K. N., Rashmi V., Odhav B. (2013). Review on natural coumarin lead compounds for their pharmacological activity. *BioMed Research International*.

[28] He H., Zhu W., Shen Y., Yang X., Zuo G., Ha X. (2000). Flavonoid glycosides from *Clausena excavata*. *Acta Botanica Yunnanica*.

[29] Sakong P., Khampitak T., Cha'on U., Pinitsoontorn C., Sriboonlue S., Yongvanit P., Boonsiri P. (2011). Antioxidant activity and bioactive phytochemical contents of traditional medicinal plants in Northeast Thailand. *Journal of Medicinal Plant Research*.

[30] Su C.-R., Yeh S. F., Liu C. M., Damu A. G., Kuo T.-H., Chiang P.-C., Bastow K. F., Lee K.-H., Wu T.-S. (2009). Anti-HBV and cytotoxic activities of pyranocoumarin derivatives. *Bioorganic and Medicinal Chemistry*.

[31] Moure A., Franco D., Sineiro J., Domínguez H., Núñez M. J., Lema J. M. (2000). Evaluation of extracts from *Gevuina avellana* hulls as antioxidants. *Journal of Agricultural and Food Chemistry*.

[32] Jayaprakasha G. K., Girennavar B., Patil B. S. (2008). Radical scavenging activities of Rio Red grapefruits and Sour orange fruit extracts in different in vitro model systems. *Bioresource Technology*.

[33] Velioglu Y. S., Mazza G., Gao L., Oomah B. D. (1998). Antioxidant activity and total polyphenolics in selected fruits, vegetables, and grain products. *Journal of Agricultural and Food Chemistry*.

[34] Harborne J. B., Williams C. A. (2000). Advances in flavonoid research since 1992. *Phytochemistry*.

[35] Hossain M. A., Shah M. D. (2011). A study on the total phenols content and antioxidant activity of essential oil and different solvent extracts of endemic plant *Merremia borneensis*. *Arabian Journal of Chemistry*.

[36] Ekeanyanwu R. C., Njoku O. U. (2014). Acute and subacute oral toxicity study on the flavonoid rich fraction of Monodoratenui folia seed in albino rats. *Asian Pacific Journal of Tropical Biomedicine*.

[37] Mogana R., Teng-Jin K., Wiart C. (2013). Anti-inflammatory, anticholinesterase, and antioxidant potential of scopoletin isolated from *Canarium patentinervium* Miq. (Burseraceae Kunth). *Evidence-based Complementary and Alternative Medicine*.

[38] Calderón-Montaño J. M., Burgos-Morón E., Pérez-Guerrero C., López-Lázaro M. (2011). A review on the dietary flavonoid kaempferol. *Mini-Reviews in Medicinal Chemistry*.

[39] Krishnappa P., Venkatarangaiah K., Venkatesh, Rajanna S. K. S., Gupta R. K. P. (2014). Antioxidant and prophylactic effects of *Delonixelata* L., stem bark extracts, and flavonoid isolated quercetin against carbon tetrachloride-induced hepatotoxicity in rats. *BioMed Research International*.

[40] Ecobichon D. J., Hollinger M. A. (1995). Acute toxicity studies. *The Basic of Toxicity Testing*.

[41] Teo S., Stirling D., Thomas S., Hoberman A., Kiorpes A., Khetani V. (2002). A 90-day oral gavage toxicity study of d-methylphenidate and d, l-methylphenidate in Sprague Dawley rats. *Tox*.

[42] Mohamed E. A. H., Lim C. P., Ebrika O. S., Asmawi M. Z., Sadikun A., Yam M. F. (2011). Toxicity evaluation of a standardised 50% ethanol extract of *Orthosiphon stamineus*. *Journal of Ethnopharmacology*.

[43] Rhiouani H., El-Hilaly J., Israili Z. H., Lyoussi B. (2008). Acute and sub-chronic toxicity of an aqueous extract of the leaves of *Herniaria glabra* in rodents. *Journal of Ethnopharmacology*.

[44] Ashafa A. O. T., Yakubu M. T., Grierson D. S., Afolayan A. J. (2009). Toxicological evaluation of the aqueous extract of *Felicia muricata* Thunb. leaves in Wistar rats. *African Journal of Biotechnology*.

[45] Baker F. J., Silverton R. E., Pallister C. J. (2001). *Introduction to Medical Laboratory Technology*.

